# Functional (Re)Development of SYNTAX Score II 2020: Predictive Performance and Risk Assessment

**DOI:** 10.3390/jcm12185844

**Published:** 2023-09-08

**Authors:** Antonella Scala, Andrea Erriquez, Filippo Maria Verardi, Andrea Marrone, Ennio Scollo, Michele Trichilo, Alessandro Durante, Delio Tedeschi, Bernardo Cortese, Alfonso Ielasi, Giuliano Valentini, Matteo Tebaldi, Gianluca Campo, Rita Pavasini, Simone Biscaglia

**Affiliations:** 1 Cardiology Unit, Azienda Ospedaliero-Universitaria di Ferrara, 44124 Cona, Italy; antonellascala95@gmail.com (A.S.); andrea.erriquez90@gmail.com (A.E.); filippomaria.verardi@gmail.com (F.M.V.); marrone.andrea@outlook.it (A.M.); ennio.scollo@tiscali.it (E.S.); michele.trichilo@edu.unife.it (M.T.); tblmtt@unife.it (M.T.); cmpglc@unife.it (G.C.); simone.biscaglia@gmail.com (S.B.); 2 Cardiology Department, Policlinico San Marco, 24046 Zingonia, Italy; durante.alessandro@gmail.com; 3 Cardiology Department, Istituto Clinico S. Anna, 25127 Brescia, Italy; deliotds@yahoo.it; 4 Cardiology Department, Clinica San Carlo, 20037 Paderno Dugnano, Italy; bcortese@gmail.com; 5 Cardiology Department, Istituto Clinico Sant’Ambrogio, 20161 Milano, Italy; aielasi@hotmail.com; 6 Cardiology Department, Ospedale San Filippo e Nicola, 67051 Avezzano, Italy; gvalentini@asl1abruzzo.it

**Keywords:** syntax score, fractional flow reserve, multivessel disease

## Abstract

The present study investigates the prognostic value of the Syntax Score II 2020 corrected for flow-limiting lesions and its ability to better address treatment by benefit prediction among patients with left main or multivessel disease. We analyzed 1274 patients from the HALE-BOPP cohort and integrated the Syntax Score II 2020 with the result of the fractional flow reserve (FFR) evaluation. Absolute risk difference (ARD) between surgical and percutaneous revascularization was calculated for anatomic and functional Syntax Score II 2020 predicted mortality. The ARD allowed to stratify the population into two large categories: “coronary artery bypass graft (CABG) better” with ARD ≥ 4.5% and “CABG–percutaneous coronary intervention (PCI) equipoise” with ARD < 4.5%. The mean global anatomical Syntax Score was 15.5 ± 9.2, whereas the functional one was 9.5 ± 10 (*p* < 0.01). Using the anatomic Syntax Score II 2020, 881 patients had a CABG-PCI equipoise. This number increased to 1041 after considering only flow-limiting lesions by FFR (*p* < 0.001); therefore, 40% of CABG better patients were reclassified within the CABG-PCI equipoise category. Kaplan–Maier curves showed similar actual survival rates for patients originally with CABG-PCI equipoise and those reclassified, in both cases higher than those from CABG better patients (*p* < 0.01). The integration between Syntax Score II 2020 and physiology is feasible, and merging clinical, anatomic and functional data allows for better risk prediction and therapeutic guidance.

## 1. Introduction

Current guidelines recommend a heart team discussion to establish a revascularization strategy in patients with multivessel coronary artery disease (MVD) and/or left main (LM) coronary disease [1,2]. Overall, data emerging from clinical trials are all in the direction of the apparent superiority of surgical revascularization in complex anatomy settings [3,4,5]. To standardize the evaluation of coronary artery disease (CAD) complexity, the Synergy between PCI with Taxus and Cardiac Surgery (Syntax) Score was developed and validated. Initially, the focus was placed on the anatomical (and, therefore, procedural) complexity of coronary artery disease (Syntax Score) [6,7,8], while subsequently on the interaction of the clinical characteristics and comorbidities (Syntax Score II) [9]. Lastly, the Syntax Score II 2020 [10] was developed and externally validated for patients with de novo 3-vessel disease and/or LM disease, with the purpose of predicting 5- and 10-year mortality rates and 5-year major adverse cardiac and cerebrovascular events (MACE) rates for both revascularization strategies, and their absolute risk difference (ARD) [11].

The Syntax Score II 2020, however, fails to include the physiological impact of the lesions. The consequent risk is to overestimate the anatomic burden of disease by including vessels with negative physiology whose treatment does not provide the patient with a prognostic benefit. The aim of the present study is to test and investigate the clinical prognostic value of the newly (re)developed functional Syntax Score II 2020 score and to evaluate its ability to better address treatment by benefit prediction among patients with complex CAD enrolled in a prospective international registry of consecutive patients receiving FFR evaluation [12].

## 2. Materials and Methods

A detailed description of the Clinical Outcome of FFR-guided Revascularization Strategy of Coronary Lesions (HALE-BOPP) registry has been previously published [12]. In brief, the HALE-BOPP [12] is an investigator-initiated, multicenter, international prospective study with the aim to compare, at vessel-level and patient-level, the long-term outcome of FFR-based deferral vs. FFR-guided and angio-guided revascularization. The study consecutively enrolled 1305 patients who underwent FFR measurement on at least one vessel with COMET^®^ wire (Boston Scientific, St. Paul, MN, USA). Exclusion criteria were a life expectancy of less than one year because of known non-cardiovascular comorbidity, inability to guarantee clinical follow-up, and unwillingness to provide written informed consent. Patients with prior coronary artery bypass (CABG) and chronic total occlusion (CTO) were also excluded. All vessels showing a lesion with a diameter stenosis (DS) ≥ 50% (by visual estimation) were of interest to the study (2543 vessels). The vessels showing a coronary lesion with DS ≥ 90% or being the culprit lesion of an acute event must be directly treated with revascularization. The remaining coronary lesions should undergo FFR measurement to guide revascularization [12]. Operators proceeded with angio-guided revascularization in 760 vessels. Conversely, in 1662 vessels, FFR was assessed to guide revascularization. FFR value was considered flow-limiting (positive) if ≤0.80, and coronary revascularization was mandated by protocol. Overall, in 1126 (67%) vessels, the treatment was FFR-deferred, whereas 536 (33%) vessels were treated with FFR-guided PCI. The study was conducted in accordance with the ethical principles of the Declaration of Helsinki. All patients gave informed written consent, and the study was registered at ClinicalTrials.gov (NCT03079739) and approved by the ethical review boards at the participating hospitals. For the aim of the present study, we analyzed the HALE-BOPP cohort and calculated the values of anatomic and functional Syntax Score II 2020.

Anatomic and functional Syntax Score II 2020 were calculated by an independent core lab blinded to outcome data. The Syntax Score II 2020 was calculated based on anatomical data, deriving from the Syntax Score value and clinical data, as previously described [10]. To obtain the anatomic Syntax Score II 2020 value, clinical factors were associated with the anatomical data deriving from the calculation of the Syntax Score. On the contrary, functional Syntax Score II 2020 was calculated using the formula for Syntax Score II 2020, replacing the anatomic Syntax Score with the functional one [10]. Functional Syntax Score was defined as a recalculated score counting only ischemia-producing lesions as assessed by FFR (value ≤ 0.80) [9]. The 10-year mortality data was not available in the HALE-BOPP registry cohorts; therefore, validation of the Syntax Score II 2020 for all-cause mortality was performed based on the 3-year results. The same method has been used in the external validation of the FREEDOM (Strategies for multivessel revascularization in patients with diabetes), BEST (Trial of Everolimus-Eluting Stents or Bypass Surgery for Coronary Disease), and PRECOMBAT (Bypass Surgery Versus Angioplasty Using Sirolimus-Eluting Stent in Patients With Left Main Coronary Artery Disease) trials [3,4,11,13].

Based on recently published data from Hara et al. [11], the ARD threshold between the fourth and fifth group in deciles was 4.5%, which allowed to stratify the population into two large categories: “CABG better” with ARD ≥ 4.5% and “CABG-PCI equipoise” with ARD < 4.5%. The “CABG better” group had better outcomes with a surgical revascularization strategy, while the “CABG-PCI equipoise” group had similar outcomes with both revascularization strategies (surgical and percutaneous). In the present analysis, anatomic and functional Syntax Score II 2020 data were used to stratify the HALE-BOPP population in the two subgroups “CABG better” and “CABG-PCI equipoise”.

The present analysis has three main objectives. The first aim is to assess the number and percentage of patients who are reclassified by applying functional Syntax Score II 2020 in place of the anatomic one. We defined “reclassified” the group of patients moving from the “CABG better” subgroup to the “CABG-PCI equipoise” subgroup with functional vs. anatomic Syntax Score II 2020. The second aim is to validate in the HALE-BOPP cohort the prognostic stratification in subgroups (CABG better and CABG-PCI equipoise) of the anatomic Syntax Score II 2020. Finally, the third aim is to describe the prognostic stratification in subgroups (CABG better, CABG-PCI equipoise, reclassified) of the functional Syntax Score II 2020. The clinical outcome of interest was the occurrence of death. All adverse events were independently adjudicated by a blinded clinical event committee.

Descriptive statistics were performed on the overall population grouped by the study outcome. Continuous variables are presented as mean (with standard deviation) or median (with interquartile range (IQR)), according to their distribution, and categorical variables as counts and proportions (%). For continuous variables, the differences were compared between groups using the Student *t*-test and the Wilcoxon test for parametric and non-parametric data, respectively. Fisher exact or Pearson Chi-squared test, with Yate’s correction when appropriate, were employed for categorical variables comparisons. Multivariate logistic regression analysis was also used to assess independent predictors of death. The parameters analyzed in multivariate analysis were selected when the *p* value was <0.10 in the univariate analysis. Differences were considered to be statistically significant when the 2-sided *p* values were <0.05. The cumulative death rates were estimated by the Kaplan–Meier method, and comparisons of outcomes were performed using ARD with 95% CI. A 2-sided *p* value ≤ 0.05 was considered statistically significant. Analyses were performed using STATA 16 (StataCorp, College Station, TX, USA).

## 3. Results

From March 2017 to September 2019, 1305 patients were included in the HALE-BOPP study. In calculating the scores, anatomical (and then functional) Syntax Scores were missing in 30 (2%) patients. Then, the final population of the present analysis includes 1274 patients and 2422 vessels with at least an intermediate stenosis (diameter ≥ 50%) (Table 1 and Table 2). Patients were followed up for an average period of 3 years. The mean age was 68 years, 354 (28%) patients were female. Acute coronary syndrome (ACS) was the indication for the procedure in half of the sample. One-quarter of patients had either chronic kidney disease, diabetes or peripheral artery disease (PAD). The left anterior descending artery (LAD) was the most represented vessel (1112 (45%)) (Table 2), and the location of the coronary lesion was proximal in 1294 (53%) vessels (Table 2). Overall, 1562 (64%) lesions were either B2 or C (64%) according to the American College of Cardiology (ACC)/American Heart Association (AHA) classification. As anticipated, vessels showing a coronary lesion with DS ≥ 90% or being culprit lesions were directly treated with revascularization (760 vessels). Conversely, in 1126 (67%) vessels, the treatment was FFR-deferred, whereas 536 (33%) vessels were treated with FFR-guided PCI. One thousand four hundred and thirty (1430) second-generation drug-eluting stents (DES) were placed during the index procedure. Intravascular ultrasound (IVUS) was used in 143 vessels (6%) in the procedural planning.

### 3.1. Anatomic Versus Functional Syntax Score II 2020

**Figure 1:**
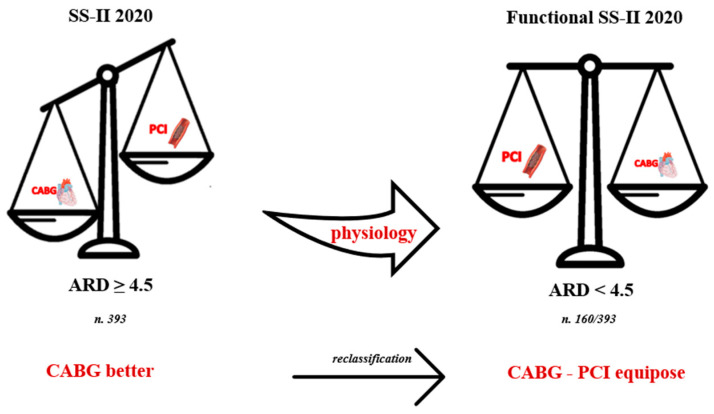
Reclassification through functional Syntax Score II 2020. One hundred sixty (40%) patients with ARD ≥ 4.5%, defined by Syntax Score II 2020, are reclassified in the “CABG-PCI equipoise” group with ARD < 4.5%. SS-II 2020: Syntax score-II-2020; ARD: absolute risk difference; CABG: coronary artery bypass graft; PCI: percutaneous coronary intervention.

**Figure 2:**
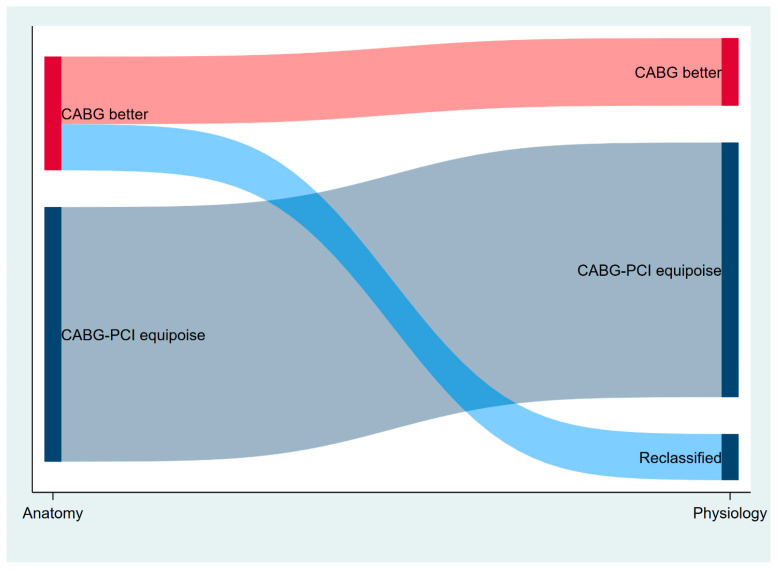
Sankey diagram showing “CABG better” patients’ reclassification into “CABG-PCI equipoise” group. CABG: coronary artery bypass graft. PCI: percutaneous coronary intervention.

The mean global anatomic Syntax Score was 15.5 ± 9.2, and the mean global functional Syntax Score was 9.5 ± 10 (*p* < 0.01). Using the anatomic Syntax Score II 2020, 881 (69%) patients were classified in the equipoise between the CABG and PCI groups. After integrating the coronary physiology result in the Syntax Score II 2020, 1041 (82%) patients were classified in the equipoise between the CABG and PCI groups. Therefore, 160 patients (40% of those with ARD ≥ 4.5%) were reclassified with the functional Syntax Score II 2020 (Figure 1 and Figure 2) (*p* < 0.001 between anatomic and functional Syntax Score II 2020).

### 3.2. Prognostic Stratification of Anatomic and Functional Syntax Score II 2020

**Figure 3:**
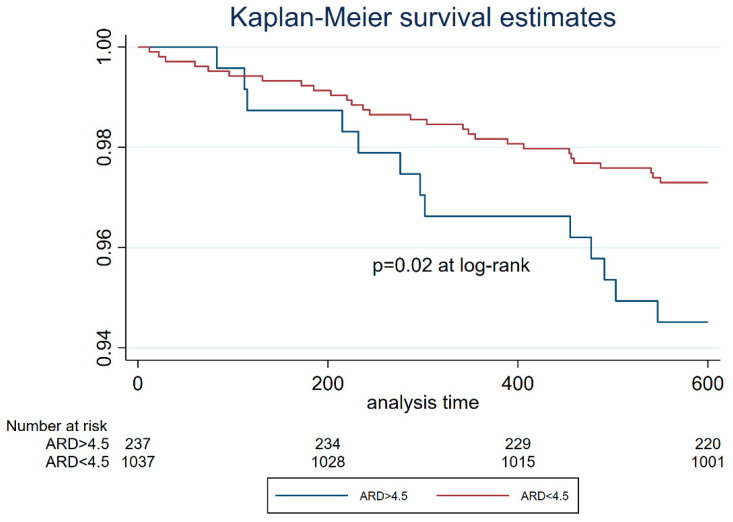
Kaplan–Meier survival estimates stratifying the study cohort based on the ARD cut-off of 4.5%. Patients with ARD ≥ 4.5%, with most comorbidities and risk factors, had the worst survival estimates, as expected. ARD: absolute risk difference.

**Figure 4:**
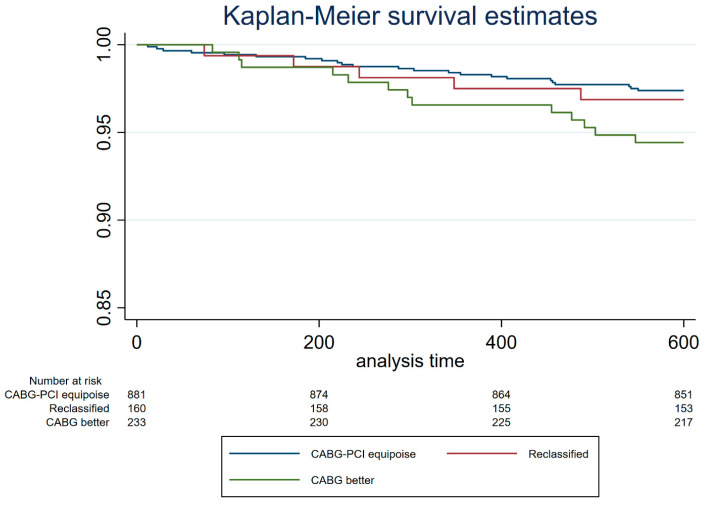
Kaplan–Meier survival estimates stratifying the study cohort based on the revascularization strategy suggested by ARD. “CABG-PCI equipoise” group is composed of patients originally with ARD < 4.5% assessed by Syntax Score II 2020. “CABG better” group is composed of patients originally with ARD ≥ 4.5%. “Reclassified” group is composed of patients reclassified using the functional Syntax Score II 2020. SS-II 2020: Syntax Score II 2020. PCI: percutaneous coronary intervention. CABG: coronary artery bypass graft.

From the univariate analysis, functional Syntax Score II 2020 resulted as one of the independent predictors of overall 3-year mortality along with age, hypertension, diabetes, COPD, PAD, LVEF, and de novo lesions (Table 3). From the multivariate analysis, LVEF, CKD, PAD and functional Syntax Score II 2020 were independent predictors of mortality (Table 3). Kaplan–Meier survival estimates based on anatomic Syntax Score II 2020 stratification showed lower survival rates for patients in the CABG better group if compared to the CABG-PCI equipoise group (*p* = 0.02, Figure 3). After the integration of physiology results into the Syntax Score II 2020, Kaplan–Meier curves showed similar survival rates for patients of the CABG-PCI equipoise and reclassified groups (Figure 4). The survival of both groups was significantly higher than that of the patients of the CABG better group (Figure 4).

## 4. Discussion

The major findings of the present study can be summarized as follows:

- The functional Syntax Score II 2020 reclassifies a significant portion of patients (≈40%) towards the equipoise between CABG and PCI, namely an ARD < 4.5% if compared to the anatomic Syntax Score II 2020.
- Kaplan–Meier curves showed no difference in survival estimates for the equipoise group based on anatomic Syntax Score II 2020 and those reclassified as equipoise after physiology assessment. In both cases, survival was greater if compared to the CABG better group.

The Syntax Score has been developed to assist and guide physicians in the choice of the correct revascularization strategy in patients with 3-vessel disease or left main disease by standardizing the evaluation of CAD anatomical complexity [7]. Afterward, the two major categories of innovation were the integration of either clinical variables or the hemodynamic impact of lesions. The first step forward was the integration of clinical variables and comorbidities with the anatomical evaluation of CAD (SS-II score and Syntax Score II 2020) [10,14]. In particular, the Syntax Score II 2020 was able to predict 5-year risk of MACE and 10-year all-cause deaths data from the SYNTAXES study with the assigned treatment (PCI versus CABG) and with two prespecified effect-modifiers (type-3 vessel disease or left main, anatomical Syntax Score) [10]. The Syntax Score II 2020 was subsequently cross-validated with the SYNTAX population and externally validated in the FREEDOM, BEST, PRECOMBAT and Hara et al. population [4,5,11,13]. In both types of validation it showed a helpful discriminative ability in both treatment groups. In parallel, another important innovation was the integration of the hemodynamic impact of lesions on purely anatomical data with the development of the functional Syntax Score to predict early and late clinical outcomes after PCI. By recalculating the score after counting only ischemia-producing lesions with FFR ≤ 0.80, the authors found that 32% of studied patients moved from higher-risk groups by Syntax Score to lower-risk groups by the functional one. The Functional Syntax Score was found not only to help stratify the risk more accurately in each patient with multivessel CAD, but it also appeared to be more closely related to prognosis after revascularization according to the risk group. [9]

The importance of functional assessment was mainly investigated and tested in the FAME series, which briefly found that physiology-guided PCI was associated with a lower rate of composite endpoint of death, non-fatal MI, and repeat revascularization at 1 year. Furthermore, a recent systematic review with 51,350 patients from 11 studies showed that physiology-guided PCI is associated with a lower-risk of MI and lower rates of MACE [6]. However, up to now, no study evaluated the impact of coronary physiology as a third modifier in the Syntax Score II 2020 to test its possible greater discriminatory power.

In the HALE-BOPP, a prospective and controlled real-life registry, 396 (31% of the cohort) patients would still derive greater benefit in terms of mortality from surgical revascularization, according to the anatomical Syntax Score II 2020 multiparameter score. This percentage mirrors previous studies and clinical practice, where the vast majority of MVD patients are still evaluated by angiography only [10,11]. However, when the functional Syntax Score II 2020 was applied, 40% of those patients anatomically addressed to CABG would derive similar benefits in terms of MACE and mortality from percutaneous revascularization, being reclassified in the lowest risk category (ARD < 4.5%) [11]. The same concept has been previously applied to the Syntax Score by showing that CAD complexity no longer portends a prognostic impairment when complete functional revascularization was obtained in 547 acute coronary syndrome patients [15]. The supremacy of physiology versus anatomy was also confirmed in the analysis of 607 patients of the FAME 2 trial in whom revascularization was not performed. Coronary stenoses were divided according to FFR (>0.80 vs. ≤0.80) and diameter stenosis (DS) (≥50% vs. <50%). Vessels with FFR ≤ 0.80 had a similar rate of vessel-oriented composite endpoint (VOCE) independently from the diameter stenosis, as well as vessels with FFR > 0.80 [16]. The present study confirms and reinforces those findings by applying physiology to the most advanced tool able to predict outcomes, namely Syntax Score II 2020. The reliability of physiology-based reclassification was tested by comparing the outcomes in terms of survival estimates of the reclassified low-risk population with both the PCI/CABG equipoise based on anatomy and the CABG better subgroup. Survival curves showed overlapping results between reclassified patients after physiology and patients classified at low risk according to anatomical Syntax Score II 2020. Furthermore, as expected, survival estimates from lower-risk populations (both original and reclassified) significantly positively diverged from the one of patients classified as suitable for CABG. In addition, the functional Syntax Score II 2020 was one of the independent predictors of outcome in the multivariable analysis.

These hypothesis-generating findings could have significant clinical implications on decision-making regarding the choice of revascularization strategies in patients with MVD and/or LM disease. Risk assessment of patients with complex CAD is, indeed, of paramount importance because of its influence on heart team decision-making.

In this way, by completing the last missing piece of the puzzle that is represented by the Syntax Score series with the functional (re)developed Syntax Score II 2020, a holistic and standardized approach to complex multivessel coronary artery disease could be achieved, limiting space for the unexpected and the variability dictated by expertise.

### Study Limitations

First, the study has the inherent limitation of its retrospective nature, being an analysis of the real-world but with a small cohort. Second, the available follow-up for the HALE-BOPP population is restricted to 3 years, while the mortality was originally tested in 10 years; however, previous external validation was based on shorter follow-up. Third, the comparison of samples numerically different in the building of K-M curves raises some concerns (881 CABG-PCI equipoise patients versus 160 reclassified patients versus 233 CABG better patients). Fourth, the presence of lesions of dubious angiographic significance may bias the representation of MVD patients. Fifth, in view of the impossibility of a wire-based functional evaluation in patients with CTO, these categories of MVD patients were excluded from the analysis, limiting the applicability and spread of the score. Lastly, despite its class of evidence, the use of physiological evaluation to set indication to PCI is still restricted, so the functional Syntax Score II 2020 may present an obstacle inherent in its nature.

## 5. Conclusions

Compared with the conventional Syntax Score II 2020, the functional (re)developed one, obtained by counting only flow-limiting lesions, increased the proportion of patients with multivessel CAD and/or left main coronary disease who fall into greatest survival estimates after revascularization. This reclassification did not translate into a worse outcome if compared to those patients where PCI/CABG equipoise was obtained on coronary anatomy evaluation. Large randomized controlled trials are needed to confirm the hypothesis of its potential central role in supporting decision-making on revascularization methods in complex CAD scenarios.

## Figures and Tables

**Table 1:** Study population.

	Patients (*n* = 1274)
Age, years	68 ± 10
Female, no. (%)	354 (28)
BMI, Kg/m^2^	28 ± 4
*Clinical history, no.* (%)	
Hypertension	1001 (78)
Hyperlipidemia	886 (69)
Current smoking	239 (18)
Diabetes mellitus	324 (25)
NID-DM	276 (21)
ID-DM	48 (3)
Prior IHD	472 (37)
Prior MI	320 (25)
Prior PCI	422 (33)
Prior CVA	60 (4)
Peripheral artery disease	357 (27)
COPD	71 (5)
CKD	332 (25)
*Clinical presentation, no.* (%)	
ACS	650 (50)
STEMI	169 (13)
NSTEMI	465 (36)
UA	16 (1)
CCS	655 (50)
Stress test	248 (44)
Imaging stress test	88 (36)
Positive stress test	226 (91)
LVEF, %	53 ± 9
LVEF < 40%	180 (14)
Three Vessel Disease	899 (68)
*Discharge medication, no.* (%)	
Aspirin	1280 (98)
P2Y12 inhibitors	1243 (95)
Oral anticoagulants	20 (2)
ACE inhibitors or ARB	1191 (91)
Beta blockers	1128 (86)
Statin	1189 (91)
High-dose statin	892 (75)
Ezetimibe	203 (16)

BMI: body mass index. IHD: ischemic heart disease. MI: myocardial infarction. PCI: percutaneous coronary intervention. CVA: cerebrovascular accident. COPD: chronic obstructive pulmonary disease. CKD: chronic kidney disease. ACS: acute coronary syndrome. STEMI: ST-segment elevation MI. NSTEMI: non-ST segment elevation MI. UA: unstable angina. CCS: chronic coronary syndrome. LVEF: left ventricular ejection fraction. ACE: angiotensin-converting enzyme. ARB: angiotensin 2 receptor blocker. NID-DM: non-insulin-dependent diabetes mellitus. ID-DM: insulin-dependent diabetes mellitus.

**Table 2:** Lesions characteristics.

	Lesions, *n* = 2422
*Territory, no.* (%)	
Left main	89 (4)
LAD	1112 (45)
LCx	626 (25)
RCA	595 (24)
*Lesion features*	
Type, no. (%)	
De novo	2250 (92)
Instent restenosis	167 (7)
Other	5 (<1)
Serial lesions, no. (%)	353 (14)
Location, no. (%)	
Proximal	1294 (53)
Mid	629 (26)
Distal	499 (20)
AHA/ACC classification, no. (%)	
A or B1	850 (35)
B2	1076 (44)
C	486 (20)
Severe calcification, no. (%)	305 (12)
Bifurcation, no. (%)	510 (21)
Severe tortuosity, no. (%)	96 (3)
*Quantitative coronary analysis*	
RVD, mm	2.6 ± 1.2
Diameter stenosis, %	66 ± 18
Lesion length, mm	15 ± 12
MLD, mm	1.27 ± 1.1
Global anatomical Syntax Score	15.5 ± 9.2
Global functional Syntax Score	9.5 ± 10
Global Syntax Score II	33.9 ± 12.5
Global functional Syntax Score II	32.2 ± 12.3
SS-II 2020, predicted MACE, PCI, %	26.1 ± 19
SS-II 2020, predicted mortality, PCI, %	37.4 ± 27.3
Functional SS-II 2020, predicted MACE, PCI, %	23.8 ± 17.9
Functional SS-II 2020, predicted mortality, PCI, %	35.3 ± 26.6

LAD: left anterior descending. LCx: left circumflex. RCA: right coronary artery. AHA: American Heart Association. ACC: American College of Cardiology. RVD: reference vessel diameter. MLD: minimal lumen diameter. SS-II 2020: Syntax Score II 2020. PCI: percutaneous coronary intervention.

**Table 3:** Univariate and multivariable analyses.

	Univariate			Multivariable		
	OR	95% CI	*p*-Value	OR	95% CI	*p*-Value
Age	1.08	1.06–1.11	<0.0001	1.04	0.99–1.08	0.054
LM-LAD versus others	0.89	0.57–1.37	0.59			
MVD	1.49	0.92–2.42	0.10	1.42	0.85–2.37	0.18
Gender	0.79	0.48–1.30	0.35			
BMI	0.99	0.98–1.01	0.21			
Hypertension	2.46	1.30–4.67	0.006	1.59	0.81–3.15	0.18
Dyslipidemia	1.23	0.77–1.94	0.39			
Smoking status:						
Current	1.29	0.81–2.04	0.29			
Former	0.90	0.49–1.63	0.72			
Diabetes	1.74	1.12–2.69	0.014			
Previous MI	1.29	0.81–2.04	0.28			
IHD	1.01	0.65–1.56	0.97			
COPD	2.23	1.10–4.50	0.026			
PAD	1.58	2.35–5.47	0.039	0.38	0.18–0.79	0.01
CKD	3.59	2.35–5.47	<0.0001	1.37	1.02–2.94	0.04
Previous stroke/TIA	1.45	0.61–3.47	0.40			
LVEF	0.95	0.93–0.97	<0.0001	0.97	0.95–0.99	0.003
Proximal disease	1.20	0.78–1.84	0.41			
% DS	1.01	0.99–1.03	0.43			
De novo vs. other	0.53	0.29–0.99	0.047	0.57	0.30–1.11	0.10
Functional SS-2020	1.03	1.02–1.03	<0.0001	1.02	1.01–1.04	0.027

OR: odds ratio; CI: confident interval; LM: left main; LAD: left anterior descendant; MVD: multivessel disease; BMI: body mass index; MI: myocardial infarction; PAD: peripheral vascular disease; IHD: ischemic heart disease; COPD: chronic obstructive pulmonary disease; TIA: transient ischemic attack; LVEF: left ventricle ejection fraction; DS: diameter of stenosis; SS: syntax score.

## Data Availability

The data underlying this article are available on reasonable request to the corresponding author.
